# Circadian Rhythms and Obesity in Mammals

**DOI:** 10.5402/2012/437198

**Published:** 2012-11-18

**Authors:** Oren Froy

**Affiliations:** Institute of Biochemistry, Food Science and Nutrition, Robert H. Smith Faculty of Agriculture, Food and Environment, The Hebrew University of Jerusalem, P.O. Box 12, 76100 Rehovot, Israel

## Abstract

Obesity has become a serious public health problem and a major risk factor for the development of illnesses, such as insulin resistance and hypertension. Attempts to understand the causes of obesity and develop new therapeutic strategies have mostly focused on caloric intake and energy expenditure. Recent studies have shown that the circadian clock controls energy homeostasis by regulating the circadian expression and/or activity of enzymes, hormones, and transport systems involved in metabolism. Moreover, disruption of circadian rhythms leads to obesity and metabolic disorders. Therefore, it is plausible that resetting of the circadian clock can be used as a new approach to attenuate obesity. Feeding regimens, such as restricted feeding (RF), calorie restriction (CR), and intermittent fasting (IF), provide a time cue and reset the circadian clock and lead to better health. In contrast, high-fat (HF) diet leads to disrupted circadian expression of metabolic factors and obesity. This paper focuses on circadian rhythms and their link to obesity.

## 1. Introduction

Obesity has become a serious and growing public health problem [1]. Attempts to develop new therapeutic strategies have mostly focused on energy expenditure and caloric intake. Recent studies link energy homeostasis to the circadian clock at the behavioral, physiological, and molecular levels [2–5], emphasizing that certain nutrients and the timing of food intake may play a significant role in weight gain [6]. Therefore, it is plausible that resetting of the circadian clock can be used as a new approach to attenuate obesity.

## 2. Circadian Rhythms 

Our planet revolves around its axis causing light and dark cycles of 24 hours. Organisms on our planet evolved to predict these cycles by developing an endogenous circadian (*circa*: about and *dies*: day) clock, which is synchronized to external time cues. This way, organisms ensure that physiological processes are carried out at the right time of the circadian cycle [7]. All aspects of physiology, including sleep-wake cycles, cardiovascular activity, endocrine system, body temperature, renal activity, gastrointestinal tract motility, and metabolism, are influenced by the circadian clock [7, 8]. Indeed, 10–20% of all cellular transcripts are cyclically expressed, most of which are tissue-specific [2, 9–13].

## 3. The Circadian Clock 

**Figure 1:**
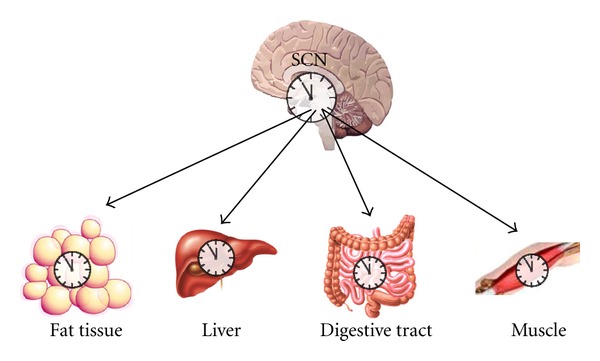
Effect of the SCN clock on peripheral clocks. The suprachiasmatic (SCN) clock resets signals in peripheral tissues, such as muscle, fat tissue, digestive tract, and liver.

The central circadian clock is located in the suprachiasmatic nuclei (SCN) of the brain anterior hypothalamus. The SCN clock is composed of multiple, single-cell oscillators synchronized to generate circadian rhythms [8, 14–16]. The endogenous period of the SCN oscillation is approximately, but not exactly, 24 h. Therefore, it requires resetting each day to the external light-dark cycle to prevent drifting out of phase. Light is a strong synchronizer for the brain clock, perceived by the retina and transmitted via the retinohypothalamic tract (RHT) to the SCN [17–19]. Similar clocks are found in peripheral tissues, such as the liver, intestine, and retina [20–22] (Figure 1). Complete destruction of the SCN abolishes circadian rhythmicity in the periphery leading to arrhythmicity [23, 24]. The SCN transmits the information to peripheral oscillators to prevent the dampening of circadian rhythms via neuronal connections or circulating factors. In turn, SCN rhythms can be altered by neuronal and endocrine inputs [25].

## 4. Physiological Effects of Reset versus Disrupted Circadian Rhythms

Disruption of circadian rhythms has negative effects on physiology. Certain pathologies, such as myocardial infarction, pulmonary edema, hypertensive crises, and asthma and allergic attacks, peak at certain times during the circadian cycle [26–28]. These findings emphasize the prominent influence of the circadian clock on human physiology and pathophysiology [8]. Living in a Western society requires us to extend wakefulness or repeatedly invert the normal sleep-wake cycle, for example, during shift work or transmeridian flights. These usually cause fatigue, disorientation, insomnia, altered nighttime melatonin levels, and hormone-related diseases [29]. Sleep disorders are also associated with impaired functioning of the central circadian clock exacerbating the disruption [8]. Disruption of circadian coordination has also been found to accelerate cancer proneness, malignant growth, and tumor progression [29–31]. Recently, the circadian clock has been linked to energy homeostasis and its disruption leads to metabolic disorders (see below). Thus, disruption of circadian coordination leads to hormone imbalance, sleep disorders, cancer proneness, and reduced life span [8, 29–33], whereas reset circadian rhythms leads to improved health and increased longevity [34–36]. Indeed, longevity in hamsters is decreased with rhythmicity disruption and is increased in old animals given fetal brain implants that restore robust rhythms [34]. Circadian rhythms also change dramatically with the age, including a shift in the phase and decrease in amplitude [20, 37–39]. 

## 5. The Molecular Clock

**Figure 2:**
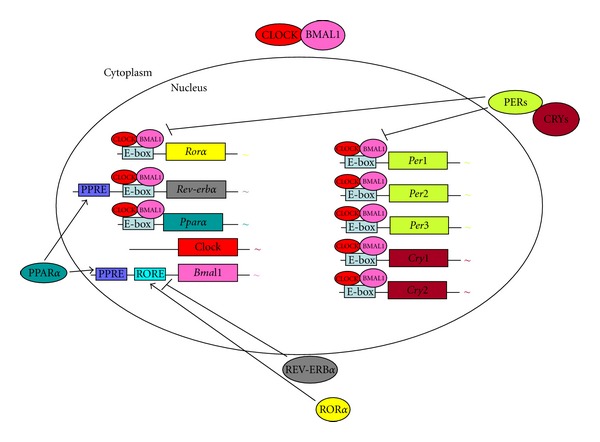
The core mechanism of the mammalian circadian clock and its link to energy metabolism. The cellular oscillator is composed of a positive limb (CLOCK and BMAL1) and a negative limb (CRYs and PERs). CLOCK and BMAL1 dimerize in the cytoplasm and translocate to the nucleus. The CLOCK:BMAL1 heterodimer then binds to enhancer (E-box) sequences located in the promoter region of *Per *and *Cry *genes, activating their transcription. After translation, PERs and CRYs undergo nuclear translocation and inhibit CLOCK:BMAL1, resulting in decreased transcription of their own genes. CLOCK:BMAL1 heterodimer also induces the transcription of *Rev-erb*α** and *Ror*α**. ROR*α* and REV-ERB*α* regulate lipid metabolism and adipogenesis, and also participate in the regulation of *Bmal1 *expression. ROR*α* stimulates and REV-ERB*α* inhibits *Bmal1 *transcription, acting through RORE. CLOCK:BMAL1 heterodimer also mediates the transcription of *Ppar*α**, a nuclear receptor involved in glucose and lipid metabolism. PPAR*α* activates transcription of *Rev-erba *by binding to a peroxisome proliferator-response element (PPRE). PPAR*α* also induces *Bmal1 *expression, acting through PPRE located in its promoter.

The circadian clock is a cellular mechanism of gene transcription, translation, and posttranslational modifications [40]. The mechanism itself exists in both the central clock and peripheral tissues. In all tissues, generation of circadian rhythms requires the coexpression of specific clock genes. The mechanism includes several key players. The transcription factor CLOCK dimerizes with BMAL1 to activate transcription upon binding to E-box (5′-CACGTG-3′) promoter elements [11]. CLOCK:BMAL1 heterodimer mediates transcription of a large number of genes including *Per*s and *Cry*s. The PERIOD proteins (PER1, PER2, and PER3) and the two CRYPTOCHROMEs (CRY1 and CRY2) operate as negative regulators [41–43]. When PERs and CRYs are produced, they oligomerize, translocate to the nucleus, and inhibit CLOCK:BMAL1-mediated transcription (Figure 2). In addition, casein kinase I epsilon(CKI*ε*) phosphorylates the PER proteins and, thereby, enhances their instability and degradation [44–46]. CKI*ε* also phosphorylates and partially activates BMAL1 [47]. 

## 6. The Circadian Clock and Metabolic Homeostasis

The circadian clock regulates metabolism and energy homeostasis in peripheral tissues [2, 48]. The expression and/or activity of certain enzymes and transport systems [49, 50] involved in the various metabolic pathways, such as cholesterol metabolism, amino acid regulation, drug and toxin metabolism, the citric acid cycle, and glycogen and glucose metabolism, exhibit circadian expression [2, 48, 51–54]. Similarly, glucose uptake and adenosine triphosphate (ATP) concentrations exhibit circadian fluctuations in brain and peripheral tissues [52, 55, 56]. Indeed, lesions of rat SCN clock abolishes daily changes in glucose homeostasis [57], altering rhythms in glucose utilization rates and hepatic glucose production. This is because the SCN projects to the preautonomic paraventricular nucleus (PVN) neurons that control hepatic glucose production [55]. 

One of the key tissues that regulate metabolism is the adipose tissue. Circadian clocks have been shown to be present in white and brown adipose tissues [58, 59]. Adipose tissue secretes metabolic mediators, such as adiponectin, resistin, visfatin, and leptin that are clock controlled [60]. In addition, key metabolic factors in adipocytes exhibit diurnal variations in expression [61]. In addition, many hormones that regulate metabolism, such as insulin [53], glucagon [62], adiponectin [60], corticosterone [63], leptin, and ghrelin [64, 65], exhibit circadian oscillation. Leptin, secreted from adipose tissue, plays an important role in appetite suppression in the brain. Plasma leptin levels are circadian with leptin peaking early in the nonactive phase, that is during the early dark phase in diurnal animals, such as monkeys and humans [66, 67], and during the early to mid-light phase in nocturnal animals, such as rats and mice [68, 69]. Neither feeding time nor adrenalectomy affects the rhythmicity of leptin release. However, ablation of the SCN eliminates leptin circadian rhythmicity in rodents, suggesting that the central circadian clock regulates leptin expression [68]. Leptin receptors are present on SCN neurons [70–72], suggesting that leptin binds directly to SCN neurons. It seems that leptin may affect the central circadian clock directly via its receptors on SCN neurons and/or through its effect on the arcuate nucleus (ARC), a region nearby the SCN involved in appetite regulation. These findings place leptin as a major bridge linking energy homeostasis and circadian control. 

## 7. Circadian Rhythms of Hormone and Metabolic Disorders

### 7.1. Insulin

Daily oscillation of insulin secretion and glucose tolerance are lost in patients with type 2 diabetes [73, 74], as are daily variations in plasma corticosterone levels and locomotor activity in streptozotocin-induced diabetic rats [75, 76]. These results suggest that loss of circadian rhythmicity of glucose metabolism may contribute to the development of metabolic disorders, such as type 2 diabetes [74–78]. 

### 7.2. Leptin

In obese subjects, leptin retains diurnal variation in release, but with lower amplitude [79, 80]. Circadian patterns of leptin concentration were distinctly different between adult women with upper-body or lower-body obesity, with a delay in peak values of leptin of approximately 3 h in women with upper-body obesity [81]. 

### 7.3. Adiponectin

The rhythmic expression of resistin and adiponectin, two cytokines secreted from adipose tissue, was greatly blunted in obese (KK) and obese, diabetic (KK-A^y^) mice [60]. In humans, circulating adiponectin levels exhibit both ultradian pulsatility and a diurnal variation. The expression of many adipokines is blunted in obese patients [68, 82, 83]. In obese subjects, adiponectin levels were significantly lower than lean controls, although the obese group had significantly higher average peak of secretion [84]. 

## 8. Mutual Regulation of Key Metabolic Factors and Clock Mechanism 

### 8.1. BMAL1

Recent molecular studies established the involvement of the activity of the positive circadian transcription factor BMAL1 in the control of adipogenesis and lipid metabolism in mature adipocytes via Wnt signaling pathway [85, 86]. Embryonic fibroblasts from *Bmal1* ^−/−^ knockout mice failed to differentiate into adipocytes. Loss of BMAL1 expression led to a significant decrease in adipogenesis and gene expression of several key adipogenic/lipogenic factors. *Bmal1* ^−/−^ mice exhibited a metabolic syndrome-like onset, that is, elevation of the level of circulating fatty acids, including triglycerides, free fatty acids, and low-density lipoprotein (LDL)-cholesterol. In addition, ectopic fat formation was observed in the liver and skeletal muscle. This could be due to loss of the functions of adipose tissue, since ectopic fat formation was not observed in tissue-specific *Bmal1* ^−/−^ mice even under high fat diet [87].Furthermore, overexpression of BMAL1 in adipocytes increased lipid synthesis activity. Thus, BMAL1, a master regulator of circadian rhythms, plays important roles in the regulation of adipose differentiation and lipogenesis in mature adipocytes [86]. These findings may explain in part why clock disruption leads to obesity. However, recently it was reported that disruption of *Bmal1*, in mice led to increased adipogenesis, adipocyte hypertrophy, and obesity, compared to wild-type mice. Attenuation of *Bmal1* function resulted in downregulation of genes in the canonical Wnt pathway, known to suppress adipogenesis and its overexpression to augmentation [85]. Clearly, BMAL1 plays a role in adipogenesis, however, more studies are merited.

### 8.2. REV-ERBs and RORs

Two other important families of factors that link the circadian clock with lipid metabolism are the REV-ERB and ROR families. REV-ERBs and RORs, which are crucial for adipocyte differentiation [88], lipogenesis and lipid storage exhibit striking circadian rhythm [61, 89]. In addition to their role in lipid metabolism and adipocyte differentiation, REV-ERBs are a negative regulator of *Bmal1* expression [90, 91]. In contrast, retinoic acid-related orphan receptor *α* (ROR*α*) is a positive regulator of *Bmal1* expression [92, 93]. In addition, the CLOCK:BMAL1 heterodimer regulates the expression of both *Rev-erb*α** and *Ror*α** [91, 92, 94] (Figure 2). Treatment of diet-induced obese mice with aREV-ERBagonist decreased obesity by reducing fat mass and markedly improving dyslipidaemia and hyperglycaemia [95], suggesting that inhibition of BMAL1 expression is beneficial for obesity (see above).

### 8.3. PPAR*α*

Peroxisome proliferator-activated receptor *α* (PPAR*α*) is a member of the nuclear receptor family. PPAR*α* serves also as a link between metabolism and the circadian clock. PPAR*α* plays a key role in the transcription of genes involved in lipid and glucose metabolism upon binding of endogenous free fatty acids [96, 97]. Its expression is mediated by the CLOCK:BMAL heterodimer. In turn, PPAR*α* binds to the peroxisome-proliferator response element (PPRE) to activate *Bmal1 *expression [4, 98, 99]. We recently showed that a PPAR*α* agonist advanced locomotor activity and feeding daily rhythms in mice [100]. 

### 8.4. PPAR*γ* Coactivator (PGC-1*α*)

PGC-1*α*, a transcriptional coactivator that regulates energy metabolism, exhibits circadian expression. In turn, PGC-1*α* stimulates the expression of *Bmal1* and *Rev-erb*α**, through coactivation of the ROR family of orphan nuclear receptors [101, 102]. The role of PGC-1*α* in the circadian system is emphasized by null mice that show abnormal diurnal rhythms of activity, body temperature, and metabolic rate, due to aberrant expression of clock genes and those involved in energy metabolism. Indeed, analyses of PGC-1*α*-deficient fibroblasts and mice with liver-specific knockdown of PGC-1*α* indicate that it is required for cell-autonomous clock function [102].

### 8.5. AMP-Activated Protein Kinase (AMPK)

AMPK is a sensor of the energy status within cells, whose activation leads to increased catabolism [103, 104]. AMPK has been found to directly phosphorylate Ser-389 of CKI*ε*, resulting in increased CKI*ε* activity leading to PERs degradation [105]. AMPK also phosphorylates and, as a result, destabilizes CRY1 in mouse fibroblasts [106]. PERs and CRYs degradation causes the relief of CLOCK:BMAL1-mediated expression leading to a phase advance in the circadian expression in some tissues [107]. Recently, it was shown that metformin, an indirect AMPK activator, leads to phase changes in a tissue-specific manner, mainly phase advances in the liver but phase delays in muscle tissue [108]. The major role of AMPK in the core clock mechanism merits further study.

### 8.6. SIRT1

Another protein found to link metabolism with the circadian clock is SIRT1, an NAD^+^-dependent histone deacetylase involved in transcriptional silencing [109, 110]. It was recently found that AMPK modulates NAD^+^ levels and SIRT1 activity [109]. Nonhistone substrates of SIRT1 include regulatory molecules that modulate energy metabolism, such as PPAR*γ* and PGC-1*α* [111], key regulators of the core molecular clock (see above). It turns out that SIRT1 interacts directly with CLOCK and deacetylates BMAL1 and PER2 [112, 113] affecting their stability. Deacetylated PER2 is further phosphorylated and degraded relieving the inhibition of CLOCK:BMAL1 heterodimer. CLOCK:BMAL1 heterodimer also regulates the circadian expression of NAMPT (nicotinamide phosphoribosyltransferase), a rate-limiting enzyme in the NAD^+^ salvage pathway. SIRT1 is recruited to the *Nampt* promoter and contributes to the circadian synthesis of its own coenzyme [114]. In addition, CLOCK and its homolog NPAS2 can bind efficiently to BMAL1 and consequently to E-box sequences in the presence of NADH and NADPH. On the other hand, NAD^+^ and NADP^+^ inhibit DNA binding of CLOCK:BMAL1 or NPAS2:BMAL1 [115, 116]. Thus, the levels of NAD^+^ together with the cycling of SIRT1 can determine the activity and robustness of clock gene transcription. 

## 9. Clock Mutants and Metabolic Disorders

Although disruption of circadian expression leads to metabolic disorders, the most compelling linkage between metabolic disorders and the circadian clock is demonstrated by the phenotypes of clock gene mutants and knockouts. 

### 9.1. Clock

Mice with a truncated exon 18 and deleted exon 19 of the *Clock* gene (*Clock* ^Δ19^ mice) have a greatly attenuated diurnal feeding rhythm, are hyperphagic and obese, and develop a metabolic syndrome of hyperleptinemia, hyperlipidemia, hepatic steatosis, and hyperglycemia [5]. However, some studies found that *Clock* mutant mice have lower serum triglyceride and free fatty acids than wild-type mice [117]. Combination of the *Clock* ^Δ19^ mutation with the leptin knockout (*ob/ob*) resulted in significantly heavier mice than the *ob/ob* phenotype [118], reiterating the contribution of clock disruption to the obese phenotype [2, 11, 48]. 

### 9.2. Bmal1

*Bmal1* ^−/−^ knockout mice, similarly to *Clock* mutant mice, exhibited suppressed diurnal variations in glucose and triglycerides as well as abolished gluconeogenesis. Although recovery from insulin-induced hypoglycemia was impaired in *Clock* mutant and *Bmal1* ^−/−^ knockout mice, the counter-regulatory response of corticosterone and glucagon was retained [119]. 

### 9.3. Per2

The diurnal feeding rhythm in *Per2* ^−/−^ mice is absent and these mice exhibit no glucocorticoid rhythm even though the corticosterone response to hypoglycemia is intact. Interestingly, although food consumption was similar during the light and dark periods, *Per2* ^−/−^ mice fed a high-fat diet developed significant obesity [120].

## 10. Effect of Restricted Feeding (RF) on Circadian Rhythms

**Figure 3:**
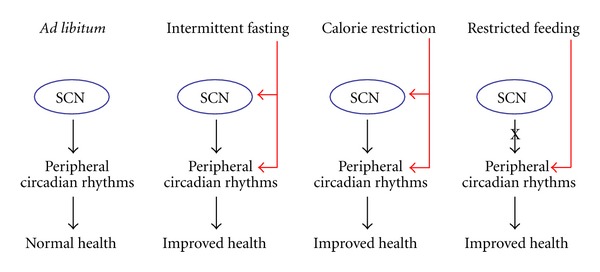
Effect of feeding regimens on circadian rhythms and health. SCN: suprachiasmatic nuclei.

RF limits the time and duration of food availability without calorie reduction, that is, food is provided *ad libitum *for about 3–5 h at the same time every day, usually at daytime [40, 49, 121, 122]. Rodents on RF, although nocturnal, adjust to the diurnal feeding period within a few days and learn to eat their daily food intake during that limited time [123–125]. Restricting food to a particular time of day has profound effects on the behavior and physiology of animals. Many physiological activities normally dictated by the SCN, such as body temperature, locomotor activity, and heart rate, are altered by RF [126–129]. 2–4 h before the meal, the animals display food anticipatory behavior, which is demonstrated by an increase in locomotor activity, body temperature, corticosterone secretion, gastrointestinal motility, and activity of digestive enzymes [122, 125, 130, 131], all are known output systems of the biological clock. RF is dominant over the SCN and is effective in all lighting conditions including in SCN-lesioned animals [122, 127, 129, 132–134]. RF affects circadian oscillators in peripheral tissues, such as liver, kidney, heart, and pancreas, with no effect on the central pacemaker in the SCN [40, 49, 121, 127, 133, 135, 136]. Thus, RF uncouples the SCN from the periphery, suggesting that nutritional regulation of clock oscillators in peripheral tissues may play a direct role in coordinating metabolic oscillations [137]. As soon as food availability returns to be *ad libitum*, the SCN clock, whose phase remains unaffected, resets the peripheral oscillators [135]. It has recently been shown that long-term day-time RF can increase the amplitude of clock gene expression, increase expression of catabolic factors, and reduce the levels of disease markers leading to better health [138] (Figure 3). Moreover, timed high-fat diet led to reduced body weight and improved metabolism compared to animals that consumed the same caloric intake spread out throughout the day [139] (see below).

Because timed feeding is dominant in resetting circadian rhythms even in animals with lesioned SCN, it has been suggested that there is a food-entrainable oscillator (FEO). However, the location of this FEO has been elusive. Lesions in brain regions involved in feeding, such as the dorsomedial hypothalamic nucleus (DMH) [140–143], the brain stem parabrachial nuclei (PBN) [140, 144], and the core and shell regions of nucleus accumbens [145, 146], revealed that these nuclei may be involved in FEO output, but they cannot fully account for the oscillation [147]. Neither vagal signals nor leptin are critical for the entrainment [130, 148]. CLOCK [149] or BMAL1 [150] and other clock genes [151] have been shown not to be necessary for food anticipatory activity. However, it has recently been demonstrated that *Per2* mutant mice did not exhibit wheel-running food anticipation [152, 153]. Thus, how RF entrains circadian rhythms remains an extremely important topic for research.

## 11. Effect of Calorie Restriction (CR) on Circadian Rhythms

CR refers to a dietary regimen low in calories without malnutrition. CR restricts the amount of calories derived from carbohydrates, fats, or proteins to 60–75% of *ad libitum*-fed animals [154]. It has been documented that calorie restriction significantly extends the life span of rodents by up to 50% [155, 156]. In addition to the increase in life span, CR also delays the occurrence of age-associated pathophysiological changes, such as cancer, diabetes, kidney disease, and cataracts [156–159]. Theories on how CR modulates aging and longevity abound, but the exact mechanism is still unknown [156]. As opposed to RF, CR entrains the clock in the SCN [160–163], indicating that calorie reduction could affect the central oscillator. CR during the daytime affects the temporal organization of the SCN clockwork and circadian outputs in mice under light/dark cycle. In addition, CR affects responses of the circadian system to light, indicating that energy metabolism modulates gating of photic inputs in mammals [164]. These findings suggest that synchronization of peripheral oscillators during CR could be achieved directly due to the temporal eating, as has been reported for RF [127, 135, 136], or by synchronizing the SCN [160–162], which, in turn, sends humoral or neuronal signals to synchronize the peripheral tissues [107, 165] (Figure 3).

## 12. Effect of Intermittent Fasting (IF) on Circadian Rhythms

During IF, food is available *ad libitum* every other day. IF-treated mice eat on the days they have access to food approximately twice as much as those having continuous access to food [166, 167]. Similarly to calorically restricted animals, IF-fed animals exhibit increased life span in comparison with the *ad libitum-*fed control [168] as well as improved glucose metabolism, cardioprotection, neuroprotection [166, 169–173], and increased resistance to cancer [167]. The IF-induced beneficial effects are thought to occur independently of the overall caloric intake, but the underlying mechanisms are still unknown. One suggested mechanism is stimulation of cellular stress pathways induced by the IF regimen [166, 174, 175]. Recently it has been shown that when food was introduced during the light period, mice exhibited almost arrhythmicity in clock gene expression in the liver. Unlike daytime feeding, nighttime feeding yielded rhythms similar to those generated during *ad libitum* feeding [176]. The fact that IF can affect circadian rhythms differently depending on the timing of food availability suggests that this regimen affects the SCN clock, similarly to CR. SCN resetting by IF and CR could be involved in the health benefits conferred by these regimens [107] (Figure 3).

## 13. Effect of High-Fat Diet on Circadian Rhythms

Few studies show that a high-fat diet leads to minimal effects on the rhythmic expression of clock genes in visceral adipose tissue and liver [177]. However, recent studies have shown that introduction of a high-fat diet to animals leads to rapid changes in both the period of locomotor activity in constant darkness and to increased food intake during the normal rest period under light-dark conditions [178]. These changes in behavioral rhythmicity correlated with disrupted clock gene expression within hypothalamus, liver, and adipose tissue, and as well as with altered cycling of hormones and nuclear hormone receptors involved in fuel utilization, such as leptin, thyroid stimulating hormone (TSH), and testosterone in mice, rats, and humans [178–183]. Furthermore, a high-fat diet modulates carbohydrate metabolism by amplifying circadian variation in glucose tolerance and insulin sensitivity [119].

In addition to the disruption of clock gene expression, high-fat diet induced a phase delay in clock and clock-controlled genes [179, 180]. As mentioned above, AMPK activation leads to CKI*ε* activity, degradation of PERs, and to a phase advance. As the levels of AMPK decline under HF diet [179, 180], it is plausible that the changes seen in the expression phase of genes under HF diet are mediated by changes in AMPK levels. In addition to its effect on gene expression, high-fat feeding led to impaired adjustment to local time by light resetting, including slower rate of reentrainment of behavioral and body temperature rhythms after “jet-lag” tests (6 h advanced light-dark cycle) and reduced phase-advance responses to light. These results correlated with reduction in c-FOS and phosphor-ERK expression in the SCN in response to light-induced phase shifts [184].

Recently, it was shown that timed high-fat diet can prevent obesity [139, 185]. Timed HF diet led to decreased body weight, cholesterol and TNF*α* levels and improved insulin sensitivity compared with mice fed HF diet *ad libitum*. Timed HF-fed mice exhibited a better satiated and less stressed phenotype of low ghrelin and corticosterone compared with mice fed timed low-fat diet [139]. In addition, timed HF diet improved metabolic pathway function and oscillations of the circadianclockand their target gene expression. These changes in catabolic and anabolic pathways altered liver metabolome and improved nutrient utilization and energy expenditure [185]. Altogether, these findings suggest that timing can prevent obesity and rectify the harmful effects of HF diet.

## 14. Conclusion

- Western lifestyle leads to high food consumption, inactivity during the active period, enhanced activity in the rest period, and shortened sleep period.
- Disrupted biological rhythms might lead to attenuated circadian feeding rhythms, disrupted metabolism, cancer proneness, and reduced life expectancy.
- Resetting the biological clock by food or feeding time may lead to better functionality of physiological systems, preventing metabolic disorders, promoting well being, and extending life span.
- Feeding time has the ability to reset bodily rhythms.
